# Decreasing patient cost and travel time through pediatric rheumatology telemedicine visits

**DOI:** 10.1186/s12969-016-0116-2

**Published:** 2016-09-20

**Authors:** Elizabeth A. Kessler, Ashley K. Sherman, Mara L. Becker

**Affiliations:** 1 Division of Rheumatology, Children’s Mercy, Kansas City and University of Missouri, Kansas City, 2401 Gillham Road, Kansas City, MO 64108 USA; 2 Division of Health Services and Outcomes Research, Children’s Mercy, Kansas City, 2405 Grand, Kansas City, MO 64108 USA

**Keywords:** Pediatric rheumatology, Telemedicine, Cost, Financial burden

## Abstract

**Background:**

There is a critical shortage of pediatric rheumatologists in the US. Substantial travel to clinics can impose time and monetary burdens on families. The aim of this study was to evaluate the cost of in-person pediatric rheumatology visits for families and determine if telemedicine clinics resulted in time and cost savings. Factors associated with interest in telemedicine were also explored.

**Methods:**

Surveys were offered to parents and guardians of patients in Pediatric Rheumatology follow-up clinics in Kansas City, Missouri, the primary site of in-person care, and at a telemedicine outreach site 160 miles away, in Joplin, Missouri. Survey questions were asked about non-medical, out-of-pocket costs associated with the appointment and interest in a telemedicine clinic.

**Results:**

At the primary Kansas City clinic, the median distance traveled one-way was 40 miles [IQR = 18–80]. In the Joplin sample, the median distance traveled to the telemedicine clinic was 60 miles [IQR = 20–85] compared to 175 miles [IQR = 160–200] for the same cohort of patients when seen in Kansas City (*p* < 0.001). When the Joplin cohort was seen via telemedicine they missed less time from work and school (*p* = 0.028, *p* = 0.003, respectively) and a smaller percentage spent money on food compared to when they had traveled to Kansas City (*p* < 0.001). There was no statistical difference between the Joplin cohort when they had traveled to Kansas City and the Kansas City cohort in terms of miles driven to clinic, time missed from work and school, and percentage of subjects who spent money on food.

**Conclusions:**

Traditional in-person visits can result in a financial toll on families, which can be ameliorated by the use of telemedicine. Telemedicine leveled the economic burden of clinic visits so that when the Joplin cohort was seen via telemedicine, they experienced costs similar to the Kansas City cohort.

## Background

Although the subspecialty of pediatric rheumatology has grown in recent years, a critical shortage of pediatric rheumatologists remains. Several states have no pediatric rheumatology representation, and in states where present, the majority reside in academic centers located in larger, more populated cities [1]. As of July, 2015 there were eight states that did not have a pediatric rheumatologist to provide care and seven states had only one [2]. Even if a family obtains an appointment with a pediatric rheumatologist, they often have to travel a considerable distance for this care; the mean distance to the nearest pediatric rheumatologist in the U.S. in 2006 was 60 miles [1]. Frequent appointments for on-going, long-term chronic care can result in excessive time missed from work and school as well as other monetary expenses associated with travel.

Telemedicine has been suggested as a method to combat the shortage of pediatric rheumatologists and address barriers in access to care in the US [3, 4]. Telemedicine is defined as “the use of medical information exchanged from one site to another via electronic communications to improve a patient’s clinical health status” [5]. It is infrequently utilized in pediatric rheumatology; a recent study of 77 pediatric rheumatology practices in the US found that only three practices (4 %) had used telemedicine, demonstrating the underutilization of this method despite its suggested potential to improve access to care [6].

Telemedicine has been successfully implemented in a variety of other pediatric subspecialties, however. Randomized controlled studies have shown the effectiveness of telemedicine in children with mental health issues, including the provision of psychotherapy and in children with attention deficit/hyperactivity disorder [7, 8]. Health care services, such as psychology, social work, and physical and occupational therapy, which are often incorporated in the team-based care approach used in pediatric rheumatology, have been effectively delivered via telemedicine [9–13]. Telemedicine has also been successfully applied to more acute care settings, such as in level I or II nurseries, emergency departments, and in some hospitalized patients who require subspecialty care [14–16]. The American Academy of Pediatrics recently issued a policy statement advocating for the use of telemedicine to address health care access and physician workforce shortages [17].

Children with rheumatic diseases make frequent visits to the rheumatologist for ongoing care, yet limited literature describes the costs to families to attend clinic visits or the impact of telemedicine in pediatric rheumatology. The primary goal of this pilot study was to determine the financial costs to families associated with traditional, in-person pediatric rheumatology clinic visits and to evaluate if telemedicine clinic visits decrease financial obligations. A secondary goal was to assess interest in telemedicine among patients seen in the traditional clinic setting and determine factors associated with increased interest.

## Methods

A single center, multi-site cross-sectional survey study was conducted at a large Midwestern academic pediatric medical center. Surveys were offered to parents and guardians of children seen for routine follow-up care in the pediatric rheumatology clinic. All patients had a known rheumatic disease and were targeted for participation at a follow up appointment. The traditional in-person visits occurred at the Children’s Mercy-Kansas City rheumatology clinic in Kansas City, Missouri, which is the primary site for the hospital system’s inpatient and outpatient care. The pediatric rheumatology telemedicine clinic is located in Joplin, Missouri, which is approximately 160 miles from Kansas City, Missouri. Telemedicine visits were performed in accordance with the Children’s Mercy telemedicine policies and procedures. These follow up visits occurred through a live, interactive audio-visual link. Additional peripheral devices, including stethoscopes, otoscopes, and mobile cameras were all available during the exam. A nurse facilitator who underwent training on the rheumatology physical examination examined the patient in Joplin while the physician in Kansas City observed and directed the exam.

After the study was approved by the hospital institutional review board, questionnaires were distributed to parents or guardians of eligible children in both the in-person Kansas City and Joplin telemedicine groups. The Kansas City group was asked about interest in a pediatric rheumatology telemedicine clinic as well as questions pertaining to the distance traveled to the appointment, amount of work and school missed, and meal and lodging costs. Joplin subjects were given a questionnaire identical to the Kansas City group except the telemedicine interest question was excluded. The Joplin subjects were asked to answer the survey questions in relation to both the current telemedicine appointment, as well as the previous in-person Kansas City appointments attended. The questionnaires were completed in 2014–15 and all costs were given in US dollars.

Statistical analyses were conducted on patients seen in Kansas City (*n* = 256) and Joplin (*n* = 24), as well as on a subsample of the Kansas City respondents living at least 50 miles from the Kansas City clinic (*n* = 58). Analysis included comparing the responses of the Kansas City subjects with the Joplin subjects, as well as comparing the responses of the Joplin subjects when seen via telemedicine with when they had previously been seen in-person in Kansas City. A revision in survey content and incompletely answered surveys are responsible for the variable number of total responses for each question. The survey was revised primarily so that subjects were able to answer numerical questions in a free-text format, rather than choose from categorical ranges. Descriptive and inferential analyses were performed using SPSS 20 and SAS 9.4, including chi-square and McNemar’s tests for categorical variable and Wilcoxon rank sum test for continuous variables. Study data were collected and managed using REDCap electronic data capture tools hosted at Children’s Mercy Kansas City.

## Results

### Distance traveled

**Fig. 1:**
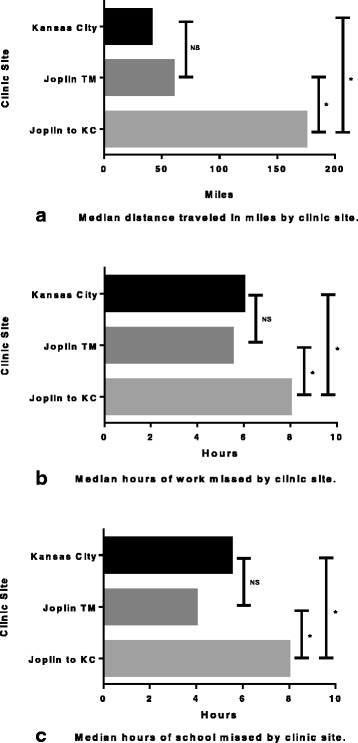
Miles traveled and time missed from work and school due to clinic visits; **a**) Distance traveled, **b**) Work missed, **c**) School missed. Joplin TM, Joplin cohort seen via telemedicine; Joplin to KC, Joplin cohort when they had previously traveled to Kansas City for clinic visits; * = *p* < 0.05; NS = not significant

The Kansas City clinic had 256 respondents. The median distance traveled one-way for Kansas City respondents was 40 miles [IQR = 18–80]. In the Joplin cohort, the median distance traveled was 60 miles [IQR = 20–85] when seen via telemedicine, which was significantly shorter than the distance traveled previously by this cohort to Kansas City (median 175 miles [IQR = 160–200], *p* < 0.001). There was no difference in the distance traveled by the Kansas City subjects compared to the Joplin cohort when seen via telemedicine (Fig. 1a).

### Time missed from work and school

Sixty two percent of Kansas City respondents missed work to take their child to the appointment and missed a median of 6.0 [4.0–8.0] hours of work. Fifty percent of Joplin respondents missed a median 5.5 [2.0–8.0] hours of work when seen via telemedicine. However, when the Joplin cohort had traveled previously to Kansas City for care, 77 % missed a median of 8.0 [8.0–8.0] hours, which is significantly more than when seen via telemedicine (*p* = 0.028). Joplin subjects missed a median of 4.0 [3.0–8.0] hours of school when seen via telemedicine in Joplin compared to 8.0 [7.0–8.0] hours for their previous appointments in Kansas City (*p* = 0.003). Neither hours spent away from work nor school were significantly different between the Kansas City respondents and the Joplin respondents when seen via telemedicine (Fig. 1b, c).

### Ancillary costs

Costs for food and lodging were also recorded. Overall, 52 % of Kansas City respondents spent money on food and 6 % spent money on lodging. Thirty eight percent of Joplin respondents spent money on meals related to the telemedicine visit in contrast to 92 % when their clinic visits occurred in Kansas City (*p* < 0.001). There was no substantial difference in percentage of patients who spent money in the Kansas City and Joplin telemedicine groups. None of the Joplin cohort had lodging expenses when attending the telemedicine clinic; however, 17 % reported this expense when traveling to Kansas City for care. Overall, Joplin respondents were more likely to spend money collectively on food, lodging and/or child care when traveling to Kansas City compared to telemedicine visits in Joplin (92 % vs. 38 %, *p* < 0.001).

### Interest in telemedicine

Of the Kansas City cohort queried, 42 % of the respondents were interested in a telemedicine option. Those who expressed interest lived further from the Kansas City clinic than those who were not interested (68 miles vs. 25 miles, *p* < 0.001). Among respondents who missed work, those who endorsed interest in telemedicine spent more hours away from work (*p* < 0.001). The number of hours of school missed and amount of money spent were not associated with increased interest in telemedicine (Table 1).

**Table 1:** Patient reported costs associated with Kansas City clinic visits and interest in telemedicine

	No interest	Interest	*p*-value
Work hours missed (median [IQR]) *n* = 84	4.0 [3.0–8.0]	8.0 [6.0–8.0]	<0.001
School hours missed (median [IQR]) *n* = 109	4.0 [2.0–8.0]	7.5 [3.5–8.0]	0.100
Amount of money spent (median [IQR]) *n* = 73	$20.00 [15–50]	$27.50 [20–50]	0.437
Miles traveled (median [IQR]) *n* = 184	25 [15–61.5]	67.5 [32.5–154]	<0.001

## Discussion

Rheumatic diseases affect an estimated 300,000 children in the United States. Despite this large number of affected children, a severe shortage of pediatric rheumatologists to diagnose and manage these patients remains. These workforce issues result in delayed diagnosis and treatment, which impedes efforts to achieve the best outcomes, which we now know result from early and effective treatment [18–20]. Telemedicine has the potential to overcome the barriers of time and distance for families and improve access to pediatric rheumatologists.

Our results are consistent with previous estimates of the average distance travelled to see a pediatric rheumatologist. The American Academy of Pediatrics estimates that about ¼ of children with rheumatic disease live 80 miles or more from a pediatric rheumatologist, which is in line with the findings in our study [21]. The median distance travelled by a family to the Kansas City clinic was 40 miles and one-quarter of our patients travelled at least 85 miles for the clinic visit. Several other pediatric subspecialists in addition to pediatric rheumatology are faced with access issues. A study examining the supply and utilization of pediatric subspecialists for 13 chronic medical conditions, one of which was arthritis, found that when parents in the lowest quintile of subspecialist supply reported an unmet need for subspecialty care, the most common barriers were related to lack of provider in the area or transportation concerns. Additionally, among all children with chronic conditions, a significantly larger percentage of children with JIA lived in areas in the lowest quintile of subspecialist supply as compared to areas in the highest quintile of supply [22]. If children with chronic arthritis are unevenly distributed, with greater numbers in areas with a decreased supply of pediatric rheumatologists, improvement in access to care for these children takes on even greater magnitude.

Because individuals with known rheumatic disease are required to make frequent visits to a rheumatologist, travelling such distances can result in the accumulation of substantial personal costs to a patient and family over time. Studies investigating the economic burden of pediatric rheumatic disease are limited, and primarily pertain to juvenile idiopathic arthritis (JIA), which is the most common rheumatic disease seen in pediatric rheumatology clinics. Results of studies are varied due to insurance differences among countries, inconsistent inclusion of patient non-health care costs, and the increasing use of biologics, which are more costly. Compared to children seen in an outpatient clinic without JIA, those with JIA had substantially higher costs related to medication use, visits to specialists and other health care providers, and diagnostic tests [23]. A Turkish study found that medications, especially tumor necrosis factor-inhibitors, accounted for about 85 % of total patient costs [24]. However, when evaluating children who were not on tumor necrosis factor-inhibitors, transportation and lodging expenses contributed to 35 % of total costs. A study conducted in Germany showed different results; transportation costs composed a majority of the out-of-pocket costs per year for a family [25]. In this cohort, 23 % of mothers and/or fathers had missed work related to their child’s JIA. This is in stark contrast to our cohort where 64 % of respondents reported missing work to attend their child’s appointment. A Nova Scotia study evaluated both patient costs as well as perceived financial burden of JIA for families [26]. Non-medical costs, specifically costs associated with visits to the tertiary care center where the rheumatology clinic was located, composed 31 % of the total costs. Annual loss of paid work accounted for another 33 % of total annual costs. Notably, the perceived financial burden of JIA was rated as either large or moderate by 36 % of the respondents and 36 % of the respondents also felt that resources to assist with costs were poor. The authors noted that even though the overall costs of having a child with JIA were modest in this study, many families still perceived the financial burden as significant and access to resources as poor.

Currently there are no published studies to our knowledge investigating the financial impact on families who utilize telemedicine in pediatric rheumatology. Even in systematic economic evaluations of telemedicine programs in general, there has been a focus on cost savings to the health care system, with less focus on financial benefits for patients. Although 29 states have mandated that commercial insurance cover telemedicine encounters, rates of reimbursement vary greatly. Despite much research indicating the efficacy of telemedicine and legislative progress, there are still reimbursement barriers related to where patients are located, what types of providers are considered eligible for reimbursement and what services are covered. The evaluation of telemedicine programs is complex and prior reviews of the literature have revealed a lack of high-quality, rigorous investigations [27, 28]. With the development of our Joplin telemedicine clinic, respondents were less likely to spend money on food, traveled a shorter distance to clinic and spent less time away from work and school compared to when they had traveled to Kansas City. Importantly, when the Joplin cohort was seen via telemedicine, this leveled the economic burden to where it was similar to the Kansas City cohort; there were no significant differences in the percentage of families who spent money on lodging, distance traveled to clinic, and the time missed from school and work between these two groups.

Despite the potential for telemedicine to improve access to care in pediatric rheumatology, it is imperative to also demonstrate that these children are receiving high quality care via this innovative method. Multiple studies in the adult rheumatology population show successful utilization of telemedicine for new patient consultations. A feasibility study in 52 new patients seen via teleconsultation where a general practitioner examined the patient while the rheumatologist observed resulted in high levels of satisfaction among the patient, general practitioner and rheumatologist. No patients required a face-to-face visit after the telemedicine consultation [29]. We have had similar satisfaction with our pediatric rheumatology telemedicine clinic. Although not part of the current study, routine surveys given to 36 patients and families at the end of rheumatology telemedicine clinic visits show that 100 % of participants would recommend a telemedicine visit to a family member or friend. When asked if visiting with the rheumatologist using telemedicine was just like the provider being in the room, 78 % strongly agreed and 22 % mostly agreed.

One of the major deficits in the tele-rheumatology literature is the lack of evidence regarding the ability to conduct a patient examination remotely and develop appropriate assessments for ongoing care [30]. A single non-randomized study of 100 new adult rheumatology referrals looked at the diagnostic accuracy in telemedicine visits. Diagnostic accuracy, which was defined as the percentage of equivalent diagnoses between a live, interactive telemedicine visit and an in-person consultation, was 97 % [31]. Notably, there have been no studies conducted in patients with known rheumatic diseases who must make frequent visits to the rheumatologist for their ongoing care, which can result in substantial financial burden to the patient over time. Certainly, the benefits of easy access are negated if the quality of care provided is subpar. Additional questions pertaining to best practices in telemedicine arise, including: how often do children with rheumatic diseases need to be seen by telemedicine, should telemedicine visits alternate with in-person visits, can the team-based approach that incorporates the services of multiple health care providers (e.g. rheumatologist, physical and occupational therapists, social worker, and psychologist) be delivered via telemedicine, and are children with certain specific rheumatic diseases better suited to be seen by telemedicine than those with other diseases? Future studies need to address these numerous questions. However, our results show that interest in this care delivery method exists and there is great potential in cost savings.

Our study has potential limitations inherent to all survey based studies. It is possible that respondents may have overestimated costs related to their rheumatology appointments. However, we do know that the reported distance to clinics by respondents is consistent with data obtained from prior studies [1, 21]. Additionally, the survey was changed during the course of the study to allow for open ended answers. This led to variable response rates among the questions depending on the ability to combine answers from different survey answers during the statistical analyses. The sample size of the Joplin telemedicine population was small, though a near 100 % response rate within this group decreases the risk of sampling error. Although this survey was conducted at a single center, which inherently limits generalizability, the clinic services four surrounding states and the population served is a mixture of rural, suburban and urban patients by the nature of the region. Finally, our study focused on time and cost savings for families related to telemedicine visits. These factors were based on our interest in these issues as drivers for interest in telemedicine. Certainly, other factors, such as money spent on clinic visits and medications, convenience or access to multidisciplinary care, insurance coverage for labs, radiology services or clinic visits themselves impact the care of a child with rheumatic disease.

## Conclusions

In conclusion, this study highlights the costs incurred by families to travel to pediatric rheumatology clinic appointments in a large Midwestern city. We have demonstrated that telemedicine is an effective way to lessen the financial burden for families that travel considerable distances for pediatric rheumatology care. Although further research is definitely needed to assess the quality of care and outcomes in children with rheumatic diseases who receive care via telemedicine, the results of this study reveal the cost savings to these patients and families and factors that contribute to interest in this innovative approach. These data will provide a foundation for future studies to address the multitude of questions that naturally arise when exploring this innovative method of providing clinical care. As we explore the most effective ways to meet the clinical need for rare pediatric subspecialties, including telemedicine in the armamentarium of options may prove to be an effective and patient centered modality for clinical care.
